# Modelling Skin Pigmentation Using the Monte Carlo Technique: A Review

**DOI:** 10.3390/s26082337

**Published:** 2026-04-10

**Authors:** Raghda Al-Halawani, Meha Qassem, Panicos A. Kyriacou

**Affiliations:** Research Centre for Biomedical Engineering, City St George’s, University of London, Northampton Square, London EC1V 0HB, UK; meha.qassem@citystgeorges.ac.uk (M.Q.); p.kyriacou@citystgeorges.ac.uk (P.A.K.)

**Keywords:** Monte Carlo modelling, skin pigmentation

## Abstract

The impact of skin pigmentation on the accuracy of optical biomedical devices has gained increased attention since the COVID-19 pandemic, particularly following evidence of oximetry measurement bias in dark-skinned individuals. Meanwhile, many computational models utilising the Monte Carlo (MC) technique have been developed as a cost-effective and scalable method for investigating these effects. Hence, this review explores the application of the MC technique in modelling skin pigmentation, focusing specifically on how melanin in the epidermis is represented across different studies. First, the biological mechanisms of pigmentation and current stratification methods are outlined to contextualise the variability in skin tone, followed by the principles of MC modelling, including photon scattering, absorption, reflection, and detection. Following a screening and exclusion process, 50 studies were evaluated in terms of how melanin concentration and distribution are incorporated into MC models and their applications, revealing a range of approaches that include analytical equations, experimental optical property measurements, or hybrid methods. The benefits and limitations of each approach is discussed, in addition to emerging advancements such as heterogeneous melanin distribution and the relation between optical properties and skin colour classification scales. Overall, the review outlines the current methodological approaches utilised for skin pigmentation modelling and offers a reference framework for researchers seeking to improve the representation of skin pigmentation in MC-based optical simulations.

## 1. Introduction

Advancements in biomedical engineering have significantly enhanced the precision, accessibility, and functionality of medical devices, with optical technologies playing a central role in diagnostics and monitoring. As the field evolves, there is growing emphasis on ensuring that devices that rely on light detection such as pulse oximetry, Near Infrared Spectroscopy (NIRS), and medical artificial intelligence (AI) and machine learning (ML) models are made more reliable across diverse populations [1,2,3]. Historically, skin pigmentation was not widely considered a factor that could alter device performance or clinical interpretation. However, a growing body of clinical evidence has suggested otherwise since the COVID-19 pandemic, revealing measurable discrepancies in optical signals potentially due to distinct differences in melanin content. Since these findings have prompted renewed investigation into how pigmentation may confound bio-optical measurements, it has challenged long-standing assumptions in both clinical practice and medical device design. As a result, researchers are now actively exploring how to better account for this variability, both in algorithm development and sensor calibration.

A promising avenue for such exploratory research lies in computational modelling, which, compared to experimental procedures, can be less resource-intensive and logistically constrained. Among the computational methods that offer a flexible and cost-effective platform to simulate light–tissue interactions under controlled conditions are Monte Carlo (MC) models. Over the past 30 years, they have emerged as a gold standard for studying photon transport in biological tissue. As a probabilistic technique, they are particularly well-suited to capturing the inherently stochastic nature of light scattering in layered and heterogeneous structures such as human skin. They are also very versatile frameworks which can be developed and utilised to predict the distribution of tissue chromophores [4], derive the optical properties of tissue layers and constituents [5], and analyse photon behaviour in complex biological structures and different sensor configurations [6]. Since skin is the largest organ in the human body, pigmentation can confound PPG signal acquisition across many biomedical applications. Therefore, it is critical to model pigmentation as accurately as the simulation framework allows. With this, the literature has revealed different modelling approaches using the Monte Carlo technique, with some prioritising anatomical realism and others computational efficiency. These varying methodologies must be critically assessed, given the growing demand of this field.

Hence, this review aims to explore the use of the Monte Carlo technique as a tool for investigating the impact of skin pigmentation in biomedical optics. It begins with a discussion on the biological basis of pigmentation and current methods used for its assessment, followed by an overview of Monte Carlo simulation principles. Next, a focused literature search is conducted to demonstrate how the technique has been employed to model pigmentation, concluding with a discussion of methodological trends, challenges, and opportunities for future research. In doing so, this review not only consolidates what has already been achieved in the field but also serves as a methodological overview of current modelling practices, providing researchers with a reference framework for selecting appropriate approaches, refining existing models, and developing novel strategies for improved representation of skin pigmentation in optical simulations.

## 2. Skin Pigmentation

### 2.1. The Production of Skin Pigmentation

The colour and appearance of skin tone arises from a complex interplay of biological components including melanin, carotene, haemoglobin, and keratin. The morphology and distribution of melanin plays a dominant role in skin colour stratification, while other chromophores modulate colouration, more so in lighter types [7]. The key microstructures involved in melanin synthesis include melanocytes, melanosomes, and keratinocytes, all of which contribute to pigmentation and skin protection.

Upon skin’s exposure to stimuli such as UV radiation or changes in hormone levels, including thyroxin, oestrogen, melanocytes, etc., which are specialised cells in the basal layer of the epidermis, respond by producing melanin. The pigment is synthesised via an enzymatic pathway involving tyrosinase, which converts the amino acid tyrosine into melanin [8]. There are two main types of melanin: eumelanin, which produces brown to black pigmentation and offers strong UV protection, and pheomelanin, which produces yellow to reddish hues and provides less UV protection [9]. The synthesis and regulation of melanin are influenced by genetic and environmental factors which make skin colour either constitutive or facultative, respectively.

**Figure 1:**
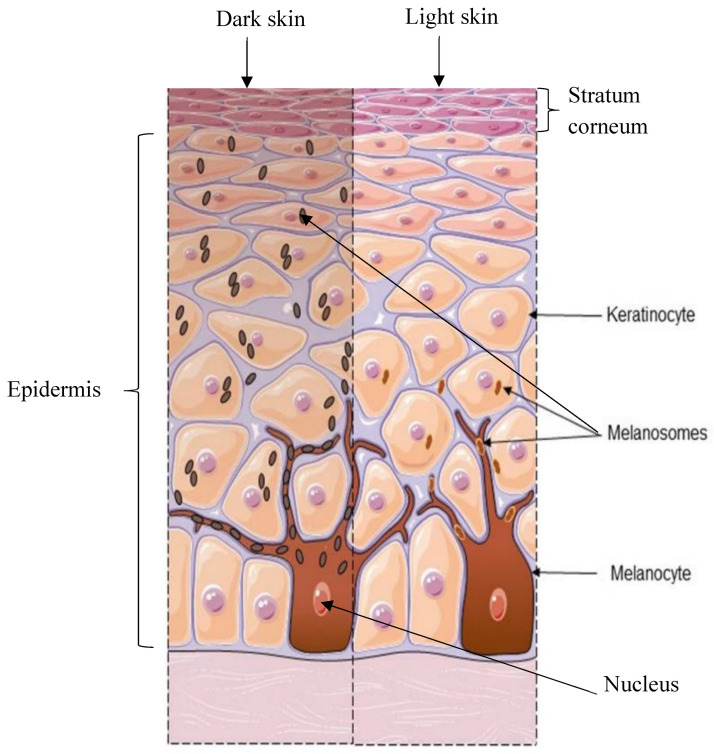
Structural components in the epidermis contributing to skin pigmentation in light and dark skin. Adapted from [11].

Moreover, different epidermal components function to form a protective barrier, regulate pigmentation, and maintain skin homeostasis (Figure 1). Melanosomes are organelles within melanocytes which store and transport melanin after it is synthesised to nearby keratinocytes. These organelles undergo maturation, gradually accumulating melanin through a cellular process of exocytosis and phagocytosis. Exocytosis involves the releasing of melanosomes from the melanocyte, and phagocytosis involves the absorption of the melanosomes by keratinocytes [10]. The melanin in these melanosomes is distributed throughout the epidermis, where it forms a protective shield around the nuclei of the keratinocytes to protect them from UV-induced DNA damage and influences the appearance of visible skin colour.

As keratinocytes proliferate through mitosis (cell division) in the basal layer, they migrate upwards towards the surface of the skin. During this process, the keratinocytes undergo a series of changes in structure and function, also known as differentiation. These changes include the accumulation of keratin, a protein that strengthens the skin, as well as the absorption of melanin from the melanocytes. As keratinocytes move towards the skin’s surface, they become flattened and eventually die, forming the stratum corneum, the outermost protective layer of dead cells, known as corneocytes. This final layer acts as a durable barrier, preventing harmful pathogens from entering the body and reducing water loss [9,11].

### 2.2. Measuring Skin Pigmentation

Accurate characterisation of skin pigmentation is critical for experimental and simulation studies, particularly for applications like pulse oximetry, where pigmentation is proposed to influence light–tissue interactions. Various qualitative and quantitative methods have been developed to stratify skin pigmentation, each with their advantages and limitations. These methods are broadly categorised as ‘subjective’ or ‘objective’ approaches depending on their underlying principles. Subjective methods rely on human perception and interpretation and include visual comparisons or colour scales. However, objective methods utilise instruments and standardised measurements such as spectrophotometers to quantify skin pigmentation. By combining both types of methods, researchers and clinicians can leverage the practicality of subjective assessments alongside the rigour of objective quantification. A comprehensive overview of the several subjective and objective methods for stratifying skin pigmentation is available on the Open Oximetry Group webpage [12], some of which are outlined in Table 1.

Among the commonly used subjective scales are the Fitzpatrick Skin Phototype Scale (FST), the Felix Von Luschan Chromatic Scale, the Munsell colour system, the Massey Scale, and the Monk Scale. The Fitzpatrick scale categorises skin into six types ranging between skin types I–VI and is widely used in dermatology. Traditionally, it is used to evaluate parameters related to predicting the skin’s reaction to ultraviolet light [13]. As a result, it may not account for all aspects of skin colour, such as undertones, redness, or colour evenness [14]. This approach risks introducing bias into the subjective stratification of skin pigmentation, which may reduce the reliability and applicability of traditional phototyping in clinical research. Although strong correlations have been observed between the melanin index (MI) and individual typology angle (ITA°) values with the Fitzpatrick scale [15], there is some evidence that criticises the use of FST in non-dermatological research. For instance, a study by He et al. showed that the patient’s self-reported race and pigmentary phenotypes are inaccurate predictors of sun sensitivity as defined by Fitzpatrick scale [16]. Moreover, another study presented by Pershing et al. employed reflectance spectrophotometry to quantify skin pigmentation in anatomical sites that are both protected and unprotected from UV radiation [17]. With a motivation to assess the correlation between objective skin pigmentation stratification methods and the Fitzpatrick scale, they found that in some cases there is a lack of complete agreement between the phenotype group assigned to the patient by the clinician and the spectra obtained.

Similarly, the Von Luschan Scale uses visual comparison against 36 coloured tiles, providing finer stratification than Fitzpatrick, but still relying on subjective interpretation and environmental lighting. Treesirichod et al. demonstrated a statistically significant correlation between skin colour evaluation using this skin colour scale chart and measurements obtained from the Mexameter MX18 (Courage & Khazaka electronic GmbH, Cologne, Germany), a narrowband reflectance spectrophotometer. They reported correlation coefficients of 0.9 and 0.86 for the melanin and erythema indices, respectively, suggesting that such colour scales can serve as practical tools for skin pigmentation stratification. In effect, this can offer a cost-effective alternative to expensive, maintenance-intensive spectrophotometric devices, even though objective methods were still deemed more reliable for precision. The study also revealed congruence between Fitzpatrick skin phototypes III–V and the Von Luschan colour ranges assessed, with increasing scale values aligning with higher Mexameter-measured indices. Future research should explore the interpersonal and intrapersonal variability within the scale and include participants across all Fitzpatrick phototypes for a comprehensive evaluation.

Moreover, some other scales including the Monk Skin Tone Scale (MST) and Massey Scale have attempted to address inclusivity gaps in older scales such as the above. The MST was developed by Dr. Ellis Monk in collaboration with Google, who divided skin into 10 shades. They used standardised photographic references in response to challenges in AI and technology systems that were often trained on biased datasets. In one study, it was reported to have the strongest correlation with variations in pulse oximetry bias when examining the impact of skin tone on SpO_2_ accuracy in critically ill patients [18]. This may show some promise in future studies, considering that the Monk Scale is one of the newest colour scales introduced in the field of skin pigmentation measurement. The Massey Scale adopts a similar principle, also focusing on pigmentation through photographic comparisons but instead under controlled lighting. It is particularly relevant for light–tissue interaction studies but is similarly constrained by observer interpretation and lighting variability. Both methods remain partly subjective and lack validation against quantitative measures, which further highlights the superiority of objective measurement of pigmentation.

As previously mentioned, the use of spectrophotometric data bridges the gap between subjective visual assessment and objective reproducible results as demonstrated by the Munsell colour system. It includes descriptions of 10 hues arranged in a circular plane to define the colour and the level of colour using 10 divisions. Another parameter, ‘value’, measures lightness or darkness on a vertical scale from 0 (black) to 10 (white), and ‘chroma’ indicates the colour’s dullness (0) or vividness (14). These are combined into a notation, such as ‘ 5R 6/12,’ which could describe a moderate red with moderate lightness and high saturation. Then, spectrophotometric devices are used to measure hue, value, and chroma to support the validation of these parameters that are selected through human assessment. This method has demonstrated higher intraclass correlation coefficients (ICC) compared to classifications based on race and ethnicity, indicating its potential as a reliable tool for quantifying skin pigmentation [19].

Lastly, methods that exclusively rely on spectrophotometry include the ITA° scale, and advanced devices such as the Konica Minolta CM-700d (Konica Minolta Ltd., Tokyo, Japan), the Dermaspectrometer (Delfin Technologies Ltd., Kuopio, Finland), and the Mexameter MX 18 as mentioned above. The ITA° scale measures the angle formed between the reflectance of skin at specific wavelengths and a baseline in the colour space, ranging from very light to dark skin tones:

(1) $$ITA{}^{\circ}=\tan^{-1}(\frac{L^{*}-50}{b^{*}})\times \frac{180}{\pi }$$

where L* represents lightness, and b* is the blue to yellow component from the L*a*b* colour space. a* is the green to red component, and although it is not considered in the equation, it is still part of the overall colour space used to define the skin colour in its entirety. Colourimeters, such as the Konica Minolta CM-700d, express skin pigmentation in colour spaces like L*a*b*; meanwhile, the Dermaspectrometer measures reflected light across a spectrum for melanin and haemoglobin.

The same spectroscopy-based measurement techniques used for objective skin-tone stratification can also be applied to extract the fundamental optical properties of skin pigmentation in the epidermis. By analysing diffuse reflectance and transmittance spectra, the absorption coefficient (μ_a_) and scattering coefficient (μ_s_) can be quantified to measure how melanin attenuates and redirects light, respectively. Furthermore, angularly resolved measurements yield the anisotropy factor (g) by using an array of photodiodes to measure angular distribution of scattered light to reveal photon directionality, and the refractive index (n) can be derived from reflectance spectra and the use of Fresnel equations. Together, these parameters allow layer-resolved modelling of how pigmentation alters photon transport through the skin.

In clinical and research contexts, choosing the appropriate tool depends on the study’s objectives. Portable devices like colourimeters are ideal for quick field assessments, while spectrophotometers are indispensable for in-depth optical analyses. By using objective systems validated against standardised metrics, bias that is inherent in visual assessments can be minimised. However, it is important to recognise that subjective methods also offer certain advantages when used appropriately and should not be entirely disregarded. These methods can be valuable in contexts where more detailed or personalised evaluation is necessary, or where objective tools may not be easily accessible. Therefore, while objective measures provide high precision, subjective scales can still have a role in skin pigmentation stratification.

**Table 1:** The different methods for stratifying skin pigmentation.

Fitzpatrick Skin Phototype Scale [20].	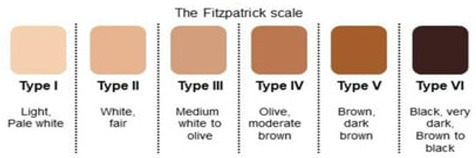
Von Luschan Chromatic Scale [12]. Red (R), Green (G), and Blue (B) values range from 0 to 255 (none to full light intensity) in the standard 8-bit system.	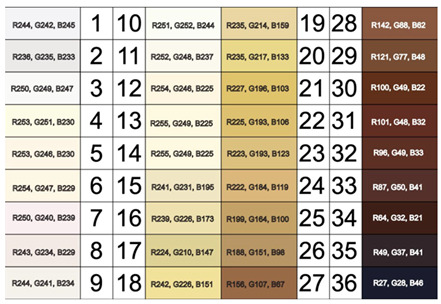
Monk Skin Tone Scale (MST Scale) [12].	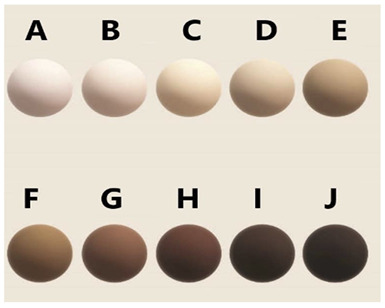
Massey Scale [12].	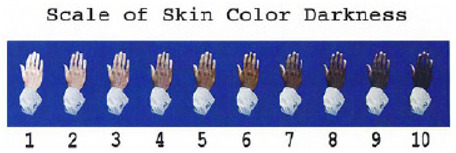
Munsell Colour chart [12]. Values with an asterisk (*) are scaled to human perception and not actual machine measurements. Y-axis = Value, x-axis = chroma.	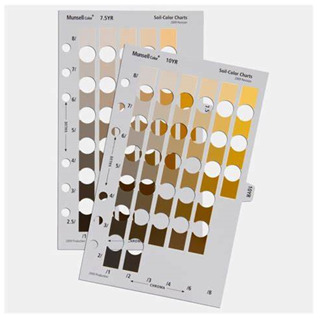
L*a*b* Scale [21]. Values with an asterisk (*) are scaled to human perception and not actual machine measurements.	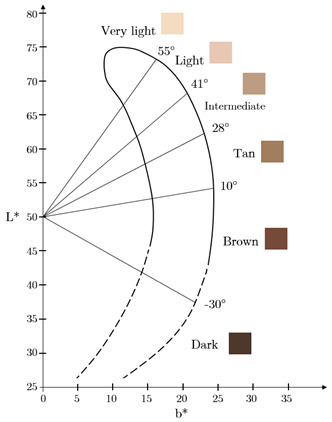
Konica Minolta CM-700d [12].	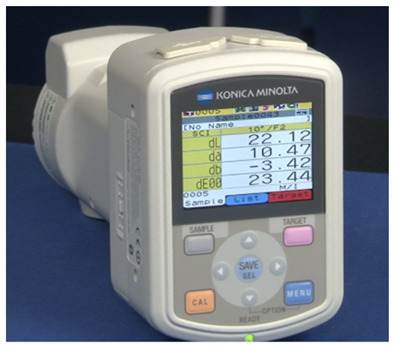
Dermaspectrometer [12]. Shows erythema index (E), melanin index (M), ITA, L*, a*, and b*.	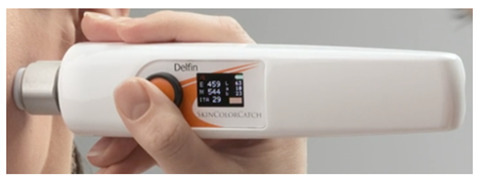
Mexameter MX 18 [12]. Show melanin index (M) and erythema index (E).	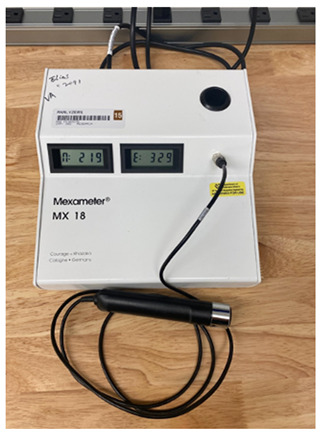

## 3. Monte Carlo Modelling

Monte Carlo models are broadly applicable in any field where predicting outcomes based on random or uncertain inputs are essential. Although they very commonly used in biomedical optics, they can also be used for predicting changes in climate change [22], operations research and logistics [23], and more. In modelling light transport in biological media, Monte Carlo techniques represent light as discrete photon packets that undergo probabilistic interactions governed by the tissue’s optical parameters (μ_a_, μ_s_, g, and n).

### 3.1. Coordinate Systems

**Figure 2:**
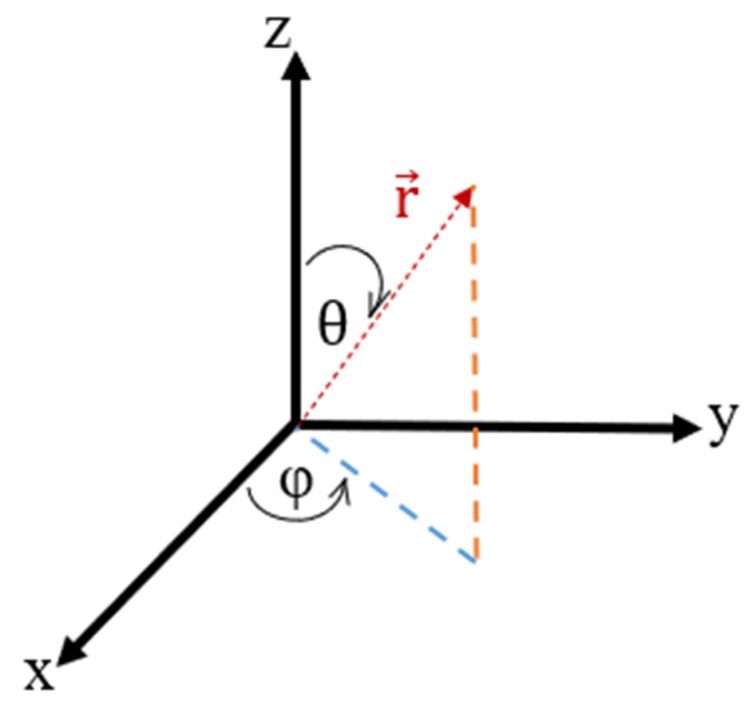
Diagrammatic representations of the Cartesian and the spherical coordinate systems. The polar (θ) and azimuthal (φ) angles are shown with respect to the x, y, z and axes in terms of vector $\vec{r}$. The blue dashed line is the projection of $\vec{r}$ onto the xy-plane, and the orange dashed line is the projection of $\vec{r}$ onto the yz-plane.

During propagation, each packet is tracked through three-dimensional space, with its position updated in a Cartesian framework (x, y, z) while changes in propagation direction due to photon scattering are described using spherical coordinates. Within the Cartesian system, the vector $\vec{r}$, which defines the propagation direction of a photon packet, forms angles α, β, and γ with the x, y, and z-axes, respectively, while, in spherical coordinates, its direction is defined by the polar angle θ, and the azimuthal angle φ (Figure 2). To translate between these coordinate descriptions, direction cosines ($u_{x,}$ $u_{y},$ $u_{z}$) are used to specify photon orientation around each axis:

(2a) $$u_{x}=\sin\left(\theta \right)\times \cos\;(\varphi )$$

(2b) $$u_{y}=\sin\left(\theta \right)\times \sin\left(\varphi \right)$$

(2c) $$u_{z}=\cos\left(\theta \right)$$

The azimuthal angle is sampled as φ=2πξ to ensure that directions are equally probable in the circular plane. ξ is a uniformly distributed random number in the interval [0, 1] using a pseudo random number generator (PRNG), meaning each value in the range has an equal chance of being selected. It commonly uses the Mersenne Twister, a deterministic algorithm in which the full sequence of values is uniquely defined by an initial seed. When the seed and configuration parameters remain unchanged, the same sequence of numbers will be reproduced exactly, ensuring computational repeatability. Hence, repeated executions of a simulation without fixing the seed will yield different numerical data, although the underlying statistical behaviour and observable relationships between inputs and outputs should remain consistent.

This combined spatial representation allows for the visualisation of light distribution within geometries commonly used to approximate tissue structures, including slabs, spheres, and cylinders. Within these frameworks, models can be constructed to investigate light–tissue interactions in a single homogeneous layer, or to consider heterogeneity by combining multiple homogeneous layers together in a specific arrangement which mimics the structure of the region of interest as much as possible. Furthermore, voxel-based representations provide an alternative to conventional dimension-defined geometries in computational modelling. In this approach, the simulated volume is discretised into a three-dimensional grid of small cubic elements, each assigned a set of local properties. Unlike analytically defined models that assume uniform properties within continuous boundaries, voxel-based methods allow properties to vary spatially, enabling the representation of more complex and heterogeneous structures. While dimension-based approaches remain computationally efficient and easier to interpret, voxel-based models provide greater flexibility and realism, albeit with increased computational and memory requirements.

### 3.2. Launching Photons

Several optical sources can be modelled in a Monte Carlo simulation, with the common ones being an optical fibre and a Gaussian beam. Depending on the optical source that is implemented in the model, the calculation for the polar angle varies and is randomly simulated. For an optical fibre, photons are emitted by considering the effective area and the reflective properties of the incident medium:

(3) $$\theta =2\times \xi \times n\times \sin^{-1}\left(NA\right)$$

where

ξ is a random number using the PRNG;
n is the refractive index of the incident medium;
NA is the numerical aperture, which determines the range of angles over which the sensor can effectively collect light.

In the case of a collimated Gaussian beam, photons are launched based on a radial probability distribution function (PDF):

(4) $$p\left(r\right)=\frac{e^{\frac{{-r}^{2}}{b^{2}}}}{{\pi b}^{2}}\times 2\pi r$$

where r is the radial distance from the centre of the beam, and b is the radius at which the intensity falls to 1e2 of its central value. In practice, photons are more likely to be densely packed near the centre of the beam, with the likelihood decreasing as you move further away from it.

The probability that a photon lies within a given radius ($r_{1}$) from the centre of the beam is then derived from the PDF, and used to set the initial x and y positions of each incident photon using trigonometric expressions:

(5a) $$r_{1}=b\times \sqrt{-ln(\xi )}$$

(5b) $$x=r_{1}\times \cos\left(\varphi \right)$$

(5c) $$y=r_{1}\times \sin\left(\varphi \right)$$

(5d) z=0

### 3.3. Photon Reflection

In Monte Carlo models, photons are simulated as clusters rather than individual photons as outlined by Wang et al. [24] and Jacques et al. [25]. They refer to this method as the ‘implicit capture’ approach and is used to improve computational efficiency and reduce variance. The initial weight of this photon cluster is equal to unity, and upon interaction with the tissue, a portion of the photon’s weight is reflected off the surface and at the boundary. The amount of the photon’s weight that is reflected is governed by Equations (6a) and (6b) depending on the angle of incidence:

(6a) $$R={\left|\frac{n_{i}\cos\left(\theta_{i}\right)-n_{t}\cos\left(\theta_{t}\right)}{n_{i}\cos(\theta_{i})+n_{t}\cos(\theta_{t})}\right|}^{2}\mathrm{for}\;\theta \neq 90{}^{\circ}$$

(6b) $${R=\left(\frac{n_{i}-n_{t}}{n_{i}+n_{t}}\right)}^{2}for\;\theta =90{}^{\circ}$$

Then, the weight of the photon entering the tissue is given by

(6c) $$w=w_{o}-(w_{o}\times R)$$

where

$w_{o}$ is the initial/previous weight of the photon cluster
w is the new weight of the photon cluster

Now the photon is ready to travel a certain step size (l). This parameter is defined as the distance that a photon travels between two consecutive interactions in the tissue, and accounts for the contributions from the absorption and scattering properties, known as the attenuation coefficient ($\mu_{t}$). The probability density function for the photon step size is given by

(7) $$p\left(l\right)={\mu_{t}\times e}^{-\mu_{t}l}$$

Similarly, the distance a photon moves ($l_{1}$) is derived from $p\left(l\right)$, such that the new position coordinates ($x_{2},y_{2},z_{2})$ are calculated:

(8a) $$l_{1}=-\frac{\ln\left(1-\xi \right)}{\mu_{t}}=-\frac{\ln\left(\xi \right)}{\mu_{t}}$$

(8b) $$x_{2}=x+l_{1}\times u_{x}$$

(8c) $$y_{2}=y+l_{1}\times u_{y}$$

(8d) $$z_{2}=z+l_{1}\times u_{z}$$

where $\mu_{t}$ is the attenuation coefficient, otherwise the sum of the absorption and scattering coefficient of the incident medium.

### 3.4. Photon Absorption and Scattering

#### 3.4.1. Scattering

In the context of biological applications, the Beer Lambert law is often modified to account for scattering effects in turbid media like tissue. To model the angular distribution of photons based on their tendency to travel in a particular direction requires the implementation of the Henyey-Greenstein phase function. This function is used to describe the relationship between the angle of deflection and the anisotropic factor (g) which determines photon directionality and ranges between −1 and 1. The equation is given by [26]:

(9a) $$p\left(cos(\theta )\right)=\frac{1-g^{2}}{4\pi {\left(1-2gcos(\theta )+g^{2}\right)}^{\frac{3}{2}}}$$

When g = −1, backscattering occurs. Rayleigh scattering occurs when g = 0 because it is isotropic, meaning that photons are deflected equally in all directions. However, when g = 1, scattering is anisotropic, which results in Mie scattering, and so deflections occur in a more forward direction.

The Henyey–Greenstein function determines the polar angle based on the scattering properties of the medium; however, the azimuthal angle is used to define the rotation of the photon around its original direction. This is also assumed to be randomly and uniformly distributed between 0 and 2π, which means that scattering has no preferred direction in the x-y plane. In Equation (9a), the ‘4π’ in the denominator accounts for all the angles around a sphere in 3D space, which is important to ensure light is scattered in all possible directions. However, in Monte Carlo simulations, photons are rotated through the azimuthal angle, which has a maximum value of 2π. To calculate a randomly generated scattering angle:

(9b) $$\cos\left(\theta )=\frac{1}{2g}[1+g^{2}-{\left(\frac{1-g^{2}}{1-g+2g\xi }\right)}^{2}\right]$$

Then, using the geometric relationship between cos (θ) and sin (θ) for a circle,

(9c) $$sin(\theta )=\sqrt{1-{cos}^{2}(\theta )},$$

the new direction cosines can be calculated after a scattering event to update the value of $u_{x}$, $u_{y}$, and $u_{z}$ [27]:

(10a) $${u_{x}}^{\prime }=\frac{\sin(\theta )\left(u_{x}u_{z}\cos\left(\varphi \right)-u_{y}\sin\left(\varphi \right)\right)}{\sqrt{1-{u_{z}}^{2}}}+u_{x}\cos\left(\theta \right)$$

(10b) $${u_{y}}^{\prime }=\frac{\sin\theta \left(u_{y}u_{z}\cos\varphi +u_{x}\sin\varphi \right)}{\sqrt{1-{u_{z}}^{2}}}+u_{y}\cos\left(\theta \right)$$

(10c) $${u_{z}}^{\prime }=-\sin\left(\theta )\cos(\varphi \right)\sqrt{1-{u_{z}}^{2}}+u_{z}\cos\left(\theta \right)$$

However, if the photons are extremely close to the z axis, the direction cosines given by Equations (2a) and (2b) are used for $u_{x1}$ and $u_{y1}$, respectively. For $u_{z1}$, this is given by Equation (2c), except that the sign of the vector is considered positive (+1) if the photon was towards the tissue surface, and negative (−1) if the photon was away from the tissue surface.

#### 3.4.2. Absorption

To model photon absorption, which is particularly important when modelling skin pigmentation, the photon’s weight is gradually reduced by employing implicit capture. This is done by subtracting a fraction (wa) of the current weight (w) of the photon after each interaction, which is characterised by the albedo (Λ): a dimensionless parameter which quantifies the dominance of absorption and scattering in a specific medium/tissue layer:

(11a) $$wa=\left(\frac{\mu_{a}}{\mu_{a}+\mu_{s}}\right)\times w=\left(\frac{\mu_{a}}{\mu_{t}}\right)\times w=\Lambda \times w$$

(11b) $$w_{new}=w-wa$$

This approach is computationally efficient, providing a smooth representation of cumulative energy deposition suitable for bulk tissue simulations. If the weight of the photon cluster does not exceed the pre-defined weight threshold of 0.0001, its intensity is considered negligible due to continuous absorption and may be terminated. To address potential bias arising from photon termination at low energies that may not contribute meaningfully to the overall simulation, a probabilistic method called Russian roulette is used. With this approach, there is a chance that a photon with a weight below the threshold will survive by checking if a random number is less than 0.1, which will increase its weight by a factor of 10, or otherwise set to zero (photon termination). When a photon is terminated, a new photon is launched, and the simulation continues until the desired number of detected photons is reached or launched. The programme then concludes once this requirement is met. In contrast, explicit capture models absorption by nullifying the weight of the photon if it experiences absorption at any interaction. This method is ideal for applications such as photothermal therapy, where understanding where photons are absorbed and how much energy is deposited is critical. Otherwise, the implicit capture approach is the ideal method for simulating photons in the model to understand the effect of skin pigmentation on bio-optical outcomes and metrics derived from PPG.

### 3.5. Photon Detection

Finally, another condition for terminating photon propagation is when the photon is detected. Detection occurs when the photon’s position and direction align with the detection area of a detector, commonly placed on the tissue surface. Depending on the setup, the detector may be positioned on the same side as the photon source, in which case reflectance is measured, or on the opposite side, where transmittance is measured. Once a photon is detected, its propagation is halted, and several variables of interest are updated. These include the total reflectance or transmittance, optical pathlength, photon weight (intensity), number of scattering events, absorbed weight in the tissue layers, distribution of scattering centres, penetration depth, etc. In transmittance geometry, the detector is positioned at (0, 0, t) where t is the thickness of the tissue, and in reflectance mode, the detector is positioned at (s, 0, 0), where s is the source–detector separation. As described in section, photon trajectories are simulated based on step sizes defined by the MC algorithm, and photons that satisfy the detection criteria are recorded by the detector. In transmissive geometry, a conditional if loop statement is used to check whether the new z coordinate ($z_{2}$) of the photon is equal to or greater than the thickness of the tissue (t) or less than 0, in reflective geometry. This determines whether the photon has been transmitted or reflected to the tissue surface (z axis), to then calculate its position in the x and y planes. The simulation runs until the desired number of photons are launched/detected, which depend on the optical, geometrical, and mechanical properties of the model.

## 4. The Use of the Monte Carlo Method for Modelling Skin Pigmentation

In order to justify and evaluate the current methodological approaches employed to incorporate skin pigmentation in such computational models, a literature search on the use of the Monte Carlo technique for modelling skin pigmentation was conducted. The following search terms were used to identify the relevant sources from Google Scholar, PubMed, Scopus, and IEEE Xplore: ‘skin pigmentation’, ‘Monte Carlo simulation’, ‘melanin’. Some of the studies that were not retrieved via the databases were also found from some of the identified publications. Studies that developed Monte Carlo models of pigmented skin in any anatomical region and published between 1995 and 2025 were considered in this review.

### 4.1. Literature Search Outcomes

A total of 401 sources were identified across four databases, including 326 from Google Scholar, 23 from PubMed, 44 from Scopus, and 8 from IEEE Xplore. Titles and abstracts were initially screened, eliminating 230 sources. Additional exclusions were made for studies not in English (3), animal-based research (7), and ocular oximetry applications (11) as they are unrelated to skin modelling, duplicates (32), and unavailable sources (10). Further removals included studies using diffuse approximation models (4) which assume a simplified light propagation approach, the Kubelka–Munk theory (1), and research employing the inverse Monte Carlo method to calculate melanin concentration (32), which is different from the forward simulations required for the studies in this thesis. Additionally, 21 theses and 1 preprint were also excluded to prioritise peer-reviewed research (22), leaving 49 sources for review and analysis. Of these studies, six maintained a constant melanin concentration or skin type ranging from light to moderate skin [6,28,29,30,31,32], but were still included as their methodological approach for representing skin pigmentation was relevant to the scope of this review. The remaining 44 sources modelled a range of skin pigmentation levels/groups, sharing some similarities in their approach [4,33,34,35,36,37,38,39,40,41,42,43,44,45,46,47,48,49,50,51,52,53,54,55,56,57,58,59,60,61,62,63,64,65,66,67,68,69,70,71,72,73,74]. A table summarising the applications and the range of skin types considered in the models are presented below in chronological order by year of publication (Table 2).

### 4.2. Optical Characterisation of the Epidermis

Accurate characterisation of epidermal optical properties is fundamental to Monte Carlo modelling of skin pigmentation. The ability to simulate light–tissue interactions depend on defining absorption and scattering coefficients and other optical properties that represent the epidermis as realistically as possible. There are three primary approaches to obtaining this data: analytical equations, experimental values from the literature, and hybrid methods that incorporate elements of both. Each approach presents distinct advantages and limitations, which must be critically evaluated to determine their applicability in each modelling framework.

#### 4.2.1. Analytical Equations

Analytical equations are mathematical formulations that are used to describe relationships based on theoretical models and empirical data from absorption spectra. From this, the absorption coefficients of dominant chromophore/s responsible for skin pigmentation are computed [6,28,30,32,33,34,39,41,42,45,46,49,53,54,55,57,63,64,68,69,70,71,72]. The epidermal absorption coefficient is at least characterised by a fraction of the absorption coefficient of melanin ($\mu_{a}melanin$) to simulate different skin types, allowing for the baseline characterisation of the epidermis without the presence of other chromophores [33,34,49,57]:

(12) $$\mu_{a}epi\left(\lambda \right)=v_{mel}\;\times \;\mu_{a}melanin\;(\lambda )$$

where $v_{mel}$ is the volume fraction of melanosomes and,

(13) $$\mu_{a}melanin\left(\lambda \right)[{mm}^{-1}]=6.6\;\times \;10^{10}{\;\times \;\lambda }^{-3.33},$$

calibrated between 300 and 1100 nm [75] and used to optically characterise melanin in a number of studies [6,30,39,46,63,65,68]. Yudovsky et al. adopted the same principle by defining the extinction coefficient of the epidermis ($\varepsilon_{mel}$) as a function of wavelength and chromophore concentration ($C_{mel}$) [54]:

(14) $$\mu_{a}epi\left(\lambda \right)=C_{mel}{\;\times \;\varepsilon }_{mel}(\lambda )$$

where $\varepsilon_{mel}$ can be calculated using the empirical equation [4,60]:

(15) $$\varepsilon_{mel}={1.7\;\times \;10}^{11}\;\times \;\lambda^{-3.48}$$

Nevertheless, accounting for absorption of light by multiple chromophores in the epidermis provides a more physiologically accurate representation of how light interacts with skin. While melanin is the dominant absorber in the visible and near-infrared spectrum, water absorption becomes significant at longer wavelengths. Neglecting water content, for example, could lead to errors in absorption calculations at wavelengths such as 940 nm, where water contributes substantially to light attenuation. Additionally, many studies report cumulative absorption coefficients for the epidermis, which inherently include contributions from water and other absorbers, such as bilirubin, carotene, etc. As a result, this creates better alignment of simulated optical property values with experimental data by utilising the following equation, adapted from Equation (12) [6,30,45,46,53,58,59,65,68,69,70,71,72]:

(16) $$\mu_{a}epi\left(\lambda \right)=v_{mel}\times \mu_{a}melanin\left(\lambda \right)+v_{water}\times \mu_{a}water\left(\lambda \right)+\left(1-v_{water}-v_{mel}\right)\times \mu_{a}baseline$$

While accurately distinguishing the baseline absorption coefficient values for a melanin-free epidermis and a blood-free dermis has been challenging, the baseline absorption for both layers is approximated. The following equation is based on measurements of bloodless rat skin using an integrating sphere calibrated with phantom measurements, also between 350 nm and 1100 nm [63,75]:

(17a) $$\mu_{a}baseline=0.0244+8.53e^{\frac{154-\lambda }{66.2}}$$

Additional data was collected between 450 nm and 750 nm using in vitro neonatal skin samples to improve the relevance of sample characterisation to human skin after accounting for excess absorption due to residual haemoglobin and bilirubin. From this data, the following baseline equation was derived [75]:

(17b) $$\mu_{a}baseline=7.84\times 10^{8}\times \lambda^{-3.255}$$

At 660 nm, skin baseline calculations are 0.0285 ${mm}^{-1}$ and 0.521 ${mm}^{-1}$, and 0.0245 ${mm}^{-1}$ and 0.165 ${mm}^{-1}$ at 940 nm, using Equation (17a) and Equation (17b), respectively. Nevertheless, these calculated baseline values account for absorption by other chromophores more accurately in comparison to assuming a constant background absorption level, as conducted by Denstedt et al., which does not account for wavelength dependence [44]:

(18) $$\mu_{a}epi\left(\lambda \right)=\mu_{a}background+v_{mel}\times \mu_{a}melanin\;(\lambda )$$

where $\mu_{a}background$ was set to 25 ${mm}^{-1}$.

The equivalent equation for multiple chromophore absorption with the extinction coefficient and concentration of a substance is [39,42,52]:

(19) $$\mu_{a}epi\left(\lambda \right)=C_{mel}\times \varepsilon_{mel}\left(\lambda \right)+C_{water}\times \varepsilon_{water}\left(\lambda \right)+\sum_{n}^{i}C_{i\ldots n}\varepsilon_{i\ldots n}\left(\lambda \right)$$

for n number of chromophores.

While the benefits of accounting for other skin chromophores have been highlighted, this approach also adds complexity, as each chromophore must be assigned a specific concentration, which can vary between individuals and anatomical locations. This could explain why Harrison-Smith et al. [56] and Arefin et al. [67] stochastically assigned a combination of different melanin, haemoglobin, and bilirubin properties to each ‘patient’ to model more realistic inter-patient variability. This way, when physical tissue samples are not accessible, more representative cohorts can be simulated to account for optical differences in skin.

Other specific equations were used to calculate the absorption coefficient of the epidermis for different applications. For instance, Larsson et al. used an equation that also follows an inverse power-law dependency on wavelength, in addition to a pre-defined proportionality constant (k) related to the specific absorption properties of melanin [55]:

(20) $$\mu_{a}epi\left(\lambda \right)=v_{mel}\times k\times ({\frac{\lambda }{\lambda_{0}})}^{-3.3}$$

where k was 39 ${mm}^{-1}$ and $\lambda_{0}$ is the reference wavelength in nanometres.

While this equation may be computationally efficient in capturing melanin’s spectral behaviour, additional work may be required to define k for other chromophores. Also, its empirical nature implies that it may not generalise well beyond certain wavelengths, with the reference wavelength being 550 nm. The equation’s inapplicability becomes more apparent for NIR tissue characterisation at even shorter wavelengths, such as UV. Huang et al. focused on modelling light–tissue interactions in this range (280 nm–400 nm) and used data from previous work to form the following equations [28]:

(21a) $$\mu_{a}epi\left(\lambda \right)=-1.125+\frac{1011}{\lambda -265.8}$$

(21b) $$\mu_{s}epi\left(\lambda \right)=\mu_{s}dermis\left(\lambda \right)=600.26{\;\times \;e}^{-0.005\lambda }$$

(21c) $$n_{epi}\left(\lambda \right)=1.395+\frac{26.57}{\lambda }+\frac{0.2203}{\lambda^{3.5}}$$

(21d) $$g_{epi}\left(\lambda \right)=0.62+2.9\times 10^{-4}\times \lambda$$

Although they do not account for changes in skin pigmentation and only Caucasian skin, these equations present the utilisation of spectra to derive equations that define the relationship between not only the absorption coefficients of the epidermis, but also the scattering coefficient, refractive index, and anisotropy factor of the epidermis. Yet, scattering is often approximated using dermal properties rather than those specific to the epidermis. This assumption can be problematic, as the structural and compositional differences between the epidermis and dermis result in distinct scattering behaviours.

Furthermore, distinguishing between eumelanin and pheomelanin absorption can provide a more detailed representation of pigmentation variability as they both have different spectral absorption and scattering properties. However, as the case with accounting for other chromophores, accurate concentrations and ratios of these components for different individuals, as well as the limited availability of spectral data for pheomelanin, may complicate the methodological approach for later analysis. These melanin components were not modelled separately in any of the studies using analytical equations for optical property classification; however, they can be considered in skin pigmentation modelling in the basal layer of the epidermis [76]:

(22a) $$\mu_{a}eu\left(\lambda \right)=6.6\times 10^{10}\times \lambda^{-3.33}$$

(22b) $$\mu_{a}pheo\left(\lambda \right)=2.9\times 10^{14}\times \lambda^{-4.75}$$

Evidently, Jacques et al. [75] characterise melanin based on brown and black colouration (Equations (13) and (16)) which can be reasonable for simulating a range of skin pigmentation levels by varying concentration. The yellow to reddish hue from pheomelanin can change the optical behaviour of the epidermis, but implementing exact concentrations of each component may be more applicable to skin cancer or UV-induced diagnoses. Overall, these equations offer a structured and theoretically grounded means of estimating optical properties, but they can be constrained by the wavelength ranges for which they were originally calibrated. From all the equations utilised in the above studies, Jacques’ equations show to be well-suited for pulse oximetry modelling, as they provide the most reliable representation of melanin’s absorption behaviour across red and infrared wavelengths.

#### 4.2.2. Spectrophotometry Measurements

An alternative approach to these mathematical formulations is the use of optical properties derived from experimental literature [29,32,35,36,37,38,40,43,47,48,50,56,61,62,66,67,73]. Empirical measurements of skin optical properties provide direct estimates of absorption and scattering coefficients, which may be considered more representative of real tissue behaviour than theoretically derived values. However, the reliability of these literature-derived properties is highly dependent on the experimental conditions under which they are obtained. Differences in measurement techniques, subject demographics, and chromophore isolation methods contribute to substantial variability in reported values. For instance, cumulative absorption coefficients can be a challenge when attempting to parameterise them separately in a Monte Carlo model, as the relative contributions of each chromophore may not be explicitly stated. However, having more than one approach to characterise tissue optically can provide a means of numerical validation. Using melanin absorption coefficient data from Alhallak [77] and Jacques equation (Equation (16)), $\mu_{a}melanin$ values can be calculated and compared at specific wavelengths. As shown in Table 3, the values from the literature and calculations are in broad agreement with each other, although comparisons with other sources can help reinforce confidence in the values to be input in the model. Similarly, optical properties that are correlated with different melanin concentration levels or skin groups may serve as a benchmark for calculated epidermal absorption coefficients [40,77] (Table 4 and Table 5).

Similar to Huang et al. study (Equations (21a)–(21d)), the scattering and anisotropy values are assumed constant for all skin levels/groups, highlighting the complexities of characterising these optical properties which rely on structural variations at the microscopic level. Hence, it is easier to obtain absorption coefficients for different skin colours because absorption is primarily dictated by the concentration and spectral properties of well-defined chromophores, making it a more direct and quantifiable process following the Beer Lambert law. Furthermore, melanin’s absorption can be measured using controlled techniques like diffuse reflectance spectroscopy [36], where the absorption spectra can be extracted from known melanin concentrations in isolated or reconstructed skin samples, which makes it possible to derive relatively consistent absorption coefficient values across different skin tones. Even so, scattering coefficient values cannot be stochastically varied based on trends, as their relationship with absorbance, transmittance, or reflectance may not follow a linear dependence on wavelength. Therefore, current Monte Carlo models may need to assume constant scattering coefficient values across all skin types until further advancements enable a more accurate representation of scattering behaviour for different levels of skin pigmentation.

#### 4.2.3. Hybrid Approach

In an attempt to address the limitations imposed by both methods, some models adopt a hybrid approach by combining analytical equations with literature-derived optical values to better define the optical properties of skin at specific wavelengths of interest and tissue layers that are not always accessible. A common example involves using calculated absorption coefficients for melanin while obtaining water absorption coefficients from experimental data [6,30]. This method leverages the strengths of both approaches by combining the flexibility of theoretical models with the empirical accuracy of measured optical properties. However, it may introduce the risk of inconsistency if values from different sources are not fully compatible with each other. For instance, if melanin absorption is estimated based on a specific assumed concentration, while water absorption is taken from a study that used different baseline conditions, the resulting cumulative optical properties may not accurately reflect real epidermal tissue. Ensuring coherence between theoretical and empirical data is therefore crucial in hybrid modelling strategies; however, this cannot always be guaranteed due to the unavailability of data from the same setup with the specific tissue components required. Establishing such a dataset would provide a more reliable source of optical property values that can be used for analytical calculations, particularly when spectrometry-based equipment is unavailable.

### 4.3. Optical Properties and Skin Classification Systems

To establish a sense of familiarity across various biomedical research fields, some studies have attempted to bridge this gap by correlating optical properties with skin classification systems such as the Fitzpatrick scale [36,37,41,43,44,45,48,52,54,59,63,64,68] and/or the individual typology angle (ITA°) based on luminance (L*) and yellow colour (b*) [73]. This approach provides a practical framework for categorising skin types based on pigmentation characteristics, enabling the assignment of optical properties to pre-defined skin groups. For instance, Ajmal et al. [43] used an epidermal absorption coefficient of 0.1865 ${mm}^{-1}$ corresponding to FST I, and 2.4668 ${mm}^{-1}$ for FST VI, which is particularly relevant in dermatological research. They further employed ITA° as an alternative classification metric, which offers a continuous rather than categorical representation of skin colour. While these classification methods facilitate structured modelling, their effectiveness depends on the accuracy of the underlying melanin concentration estimates. Since the Fitzpatrick scale was originally developed based on UV sensitivity rather than direct pigmentation measurements, its correlation with optical properties is not always straightforward but can be used as a guide for current regulations in skin stratification. Similarly, the ITA° method, while more useful for quantitative validation purposes, relies on surface reflectance measurements that do not necessarily capture deeper melanin distributions. Yet, this may not always be a priority for all applications.

## 5. Discussion

Owing to advancements in spectroscopy, Monte Carlo models have become increasingly capable of simulating a wide range of physiological parameters, enhancing their versatility in biomedical applications. By integrating spectroscopic data, these models can incorporate wavelength-dependent absorption and scattering properties of tissue and subcomponents, enabling more precise representations of skin and its influence on light propagation. This adaptability is particularly valuable in non-invasive diagnostic techniques, where subtle variations in tissue composition can significantly affect measurement accuracy. Early studies, like those by Reuss [33] and Meglinski [31], focused on melanin absorption’s impact on reflectance and SpO_2_ calibration but used limited concentration ranges. By the late 2000s, research expanded to non-invasive diagnostics and spectroscopy, incorporating broader melanin variations [4,50] and other skin chromophores such as bilirubin [32,42]. Post COVID-19, studies introduced dynamic melanin estimations [41] and addressed racial bias in device calibration [56,67]. Machine learning and optimisation techniques have further enhanced simulation accuracy and real-time analysis [57]. However, the reliability of these simulations depends on how the optical properties of the epidermis are characterised and implemented in the models.

### 5.1. Current Practices and Future Work

The current methodological approaches adopted for modelling skin pigmentation using the Monte Carlo technique offer distinct advantages and limitations. Firstly, analytical equations have shown to provide a theoretical framework for calculating absorption and scattering coefficients but are limited by the wavelength range over which they are calibrated. Many studies use similar power-law equations for melanin absorption, allowing for comparability between different research works. Secondly, literature-derived optical properties, while empirically validated, often represent averaged values that may not fully capture individual variability, which highlights the difficulty of obtaining universally comparable optical properties. Other device-related methodological differences may include the choice of light source and detection system which further complicates comparisons, as some techniques measure total attenuation rather than pure absorption. Thirdly, hybrid methods attempt to integrate the strengths of both approaches, yet inconsistencies in parameter selection can introduce uncertainties in simulations.

Beyond the inherent differences in the chemical composition of different skin types, other factors such as epidermal thickness must be considered. Changing the thickness of the epidermis also changes the probability of photons localising inside it. As a result, differences in output absorbance are likely, especially with transmittance mode pulse oximeters when used on dark-skinned individuals. In this case, the melanin index, which is a product of the epidermal thickness and the epidermal absorption coefficient, can be implemented in a Monte Carlo model [41]. A further consideration is the spatial distribution of melanin, which includes the modelling of melanosomes that are dispersed in a specific arrangement in the epidermis. From the literature, most models assumed a homogeneous melanin distribution since they are more well-suited for controlled analysis and comparison across studies, while the remainder accounted for non-uniform pigmentation patterns using voxel-based implementations [4,35,51,53,64,78]. Studies that incorporate heterogeneous melanin distributions provide a more physiologically realistic representation of skin, since melanin is not uniformly dispersed across the epidermis. However, the extent to which such complexity improves simulation accuracy must be weighed against its impact on computational efficiency, although studies investigating skin disorders like vitiligo should prioritise this type of accurate melanin distribution [4]. Nevertheless, it has been shown that physiological realism can also be achieved through stochastic sampling of different chromophore concentrations to help address the lack of data on inter-individual skin variability [56,67]. This work underscores the value of multilayer models in representing skin pigmentation, as perceived colour is influenced not only by melanin in the epidermis but also by chromophores such as blood volume in the dermis, keratin content in the stratum corneum, etc. [7]. Although this approach may impose challenges in output data interpretation as changes are no longer attributed to a single variable, it is essential for understanding how different wavelengths of light interact with the skin in MC models, as close to in vivo as possible. Other advancements can include incorporating more complex vascular structures particularly in studies of devices sensitive to blood content.

Another key challenge noted from this review is that most studies modelling skin pigmentation adopt a common strategy, which involves adjusting epidermal melanin concentration to calculate the absorption coefficient of the epidermis. Although this approach does not fully capture the physiological complexity of skin, it offers a practical means of reproducing results and comparing findings with other studies. As a result, physiological accuracy is sometimes compromised in favour of reproducibility and model validation, particularly given the inherent variability in optical property selection and tissue geometry assumptions across studies. Hence, future work should prioritise the use of open-source tools such as Monte Carlo eXtreme (MCX) or Monte Carlo Modelling of Light transport (MCML), in combination with publicly available tissue databases to provide a framework for testing hypotheses and ensuring comparability. Similarly, establishing online repositories of skin optical properties across diverse populations could reduce variability associated with literature-derived averages and encourage broader participation in simulation-based research.

### 5.2. Monte Carlo Algorithm Optimisation

Notably, current Monte Carlo studies of skin pigmentation are increasingly limited by computational demands, particularly when modelling large tissue volumes, non-homogeneous tissue layers, or multilayered tissues with distinct optical properties. Most existing simulations rely on sequential, Central Processing Unit (CPU)-based photon propagation, which is sufficient for simple layered models but becomes prohibitive as the complexity of the tissue geometry or chromophore distributions increases. Advances in high-performance computing, including open-source Graphical Processing Unit (GPU)-accelerated algorithms (e.g., MCX and Compute Unified Device Architecture (CUDA)-based implementations), can reduce runtime by orders of magnitude. Such improvements would allow researchers to simulate higher photon counts for greater statistical confidence and incorporate multiple chromophores or dynamic tissue properties without excessive computational cost. Adaptive strategies such as importance sampling may further enable a balance between computational efficiency and physiological realism by biasing the sampling toward more influential events, such as photon interaction in blood vessels for PPG signal generation, and then correcting for that bias mathematically. Additionally, the integration of data-driven approaches, such as machine learning models, can be trained on Monte Carlo outputs and act as surrogate predictors of light propagation in complex tissue geometries, enabling near real-time simulations while retaining physiologically realistic behaviours [79].

Moreover, computational optimisation does not only depend on algorithmic and methodological development, but also on the implementation of Monte Carlo algorithms in different programming environments. Historically, MC models for skin optics were developed in compiled languages such as C and C++, with the classic MCML program being written in C [80]. These languages offer the advantages of high execution speed and greater control over memory management, enabling simulations of large photon counts and complex tissue geometries with relatively low runtime. However, developing in C or C++ typically requires more extensive programming expertise which can limit accessibility for researchers without strong computational backgrounds. As a result, MATLAB, particularly versions R2016a and newer, have emerged as a popular and commonly used alternative for MC simulations, particularly in biomedical optics research, due to its extensive built-in mathematical functions and intuitive matrix operations. While it is generally slower than compiled languages for large-scale simulations, its ease of use and access to toolboxes such as the Parallel Computing Toolbox allows for multicore CPU utilisation and GPU acceleration to decrease runtime. Meanwhile, versions 3.9–3.11 of Python have also gained significant traction in recent years with its extensive scientific libraries, such as NumPy for array and matrix operations [81]. While its standalone performance remains slower than C/C++, built-in libraries such as Numba and GPU acceleration frameworks can achieve approximately 50–85% performance of optimised performance of C-CUDA [82]. This makes Python a practical option for both academic and applied research settings, especially for new users who can benefit from a large and active community and publicly access well-maintained official documentation. In essence, the choice of software implementation should be guided by the specific requirements of the study under investigation, although individuals could also benefit from the advantages of multiple programming languages simultaneously [83].

## 6. Conclusions

This review has highlighted the critical role of Monte Carlo modelling in advancing the understanding of how skin pigmentation influences light–tissue interactions in biomedical optics. It has also provided a foundation for future research aiming to refine existing models or develop new simulation strategies that better account for pigmentation-related variability in light-based biomedical technologies. By focusing on the methodological approaches used to simulate skin pigmentation computationally, it was shown that the choice of optical property characterisation can affect the accuracy and relevance of simulation outcomes. Analytical models offer theoretical consistency and reproducibility but are limited by their spectral calibration range, while experimental data provide empirical validity yet suffer from variability across sources. Hybrid methods present a promising middle ground, though they require careful harmonisation of inputs to maintain consistency. Other modelling considerations, including the distribution of melanin in the epidermis, is particularly useful in dermatological applications; however, the scattering properties of different skin pigmentation levels must be studied more rigorously. While no single approach provides a perfect solution, these advancements and considerations provide a guideline for selecting an appropriate methodological approach for modelling skin pigmentation using the Monte Carlo technique in the forthcoming studies. Ultimately, the ongoing development of such MC models must prioritise computational optimisation and spatial and optical accuracy of skin pigmentation to promote inclusivity in optical device performance.

## Figures and Tables

**Table 2:** Pertinent studies that model skin pigmentation using the Monte Carlo technique (available to access).

Study	Application	Skin Type Range	Implications of Modelling Approach
Meglinski (2001) [31]	Study how different chromophores (e.g., blood volume, oxygenation, melanin, and water content) affect skin reflectance for in non-invasive optical diagnostics.	10% melanin concentration.	Homogeneous epidermal layer neglects variation in melanin distribution, and may misrepresent how other chromophores affect reflectance.
Reuss (2005) [33]	Investigate how melanin absorption affects SpO_2_ calibration in reflectance pulse oximetry.	5%, 10%, 20% melanin concentrations.	Captures some variation in skin pigmentation, but oversimplifies distribution which may not fully reflect melanin’s impact on SpO_2_ in the specified range.
Chen et al. (2007) [4]	Investigate the effect of melanin content and distribution on skin reflectance and fluorescence spectra for non-invasive skin diagnostics, vitiligo assessment, and melanin quantification.	Normal skin: Slightly increased melanin content. Vitiligo skin: lower melanin content. Blue vitiligo skin: Melanin relocated to the dermis instead of the epidermis.	Highlights the influence of melanin localisation on spectra. Graded melanin distributions could be used to reduce bias inherent in subjective skin categories.
Ramella-Roman & Hidler (2008) [50]	Estimate skin oxygen saturation using an algorithm that accounts for melanin and haemoglobin absorption during autonomic dysreflexia events.	Skin type unspecified.	A fixed or implicit melanin absorption model is likely to underestimate how skin pigmentation affects reflectance signals since variations in melanin could alter light absorption.
Fredriksson et al. (2009) [51]	To model how melanin concentration affects Laser Doppler Flowmetry (LDF) measurements of skin microcirculation.	0% to 50% melanin concentrations, from very light to very dark skin.	Captures a very wide range of skin pigmentation levels. Back scattering light reaching the detector can underestimate microcirculatory perfusion if distribution and heterogeneity are not considered.
Swearingen et al. (2010) [42]	Improves Monte Carlo simulations for applications in non-invasive skin diagnostics, laser treatments, and optical imaging.	Different concentrations of oxyhaemoglobin, deoxyhaemoglobin, melanin, and bilirubin are varied to simulate numerous skin colour (exact number not stated).	Improves physiological representation of skin pigmentation and increases the accuracy of bio-optical outcomes across diverse skin types. Sufficient sampling of different chromophore concentrations may predict overall light–tissue interactions observed from non-homogeneous tissue layers due to the small thickness of each skin sublayer.
Maeda et al. (2010) [32]	Improve the accuracy of spectral reflectance modelling of skin tissue for skin optics research, dermatology, and cosmetics.	Exact melanin range is not stated. The study was conducted on Japanese skin, so melanin concentration can be assumed moderate (10–15%).	Ethnicity-based classification assumes uniform pigmentation within the group, overlooking significant intra-population variability as well as differences across skin groups, which can bias optical property estimation and reduce model reliability.
Liu et al. (2011) [38]	A fibre-based tissue oxygenation monitor with integrated scattering corrections to improve accuracy in skin measurements.	Volunteers categorised as white, brown, and black.	Relies on subjective, coarse groupings that do not map directly to quantitative melanin content or distribution. Can be difficult to correlate observed changes in oxygenation with skin colour.
Yudovsky et al. (2012) [54]	Separate the effects of melanin absorption in the epidermis from blood absorption in the dermis to improve tissue oxygenation measurements.	Melanin molarity: 0.01 and 0.06 μM = Light and dark skin.	Melanin molarity provides a direct, physically defined input to optical models, in comparison to melanin concentration (%) which are used to derive absorption coefficients based on empirical equations.
Okamoto et al. (2013) [66]	Investigates how melanin concentration, cosmetic powder particle size, and refractive index influence skin reflectance for cosmetic science, particularly foundation makeup for covering hyperpigmentation.	5% to 10% melanin concentrations.	Range is confined to light and moderate skin types although darker skin types (20%+) are more prone to hyperpigmentation.
Quintanar et al. (2013) [35]	Calibrate a pulse oximeter for different melanin concentrations and skin thicknesses, and photodynamic therapy.	3.8%, 13.5%, and 30.5% melanin concentration = Light, moderate, and dark pigmented, respectively.	Fixed concentrations assume homogeneous epidermal melanin and ignore particle-level effects, which can alter photon transport and result in inaccurate oxygenation readings or uneven light delivery during therapy.
Karsten & Singh (2013) [36]	Quantify the effect of epidermal absorption and thickness on fluence rate loss and adjust treatment time accordingly based on skin type.	No range specified. Uses Fitzpatrick scale (I–VI) to stratify skin colour.	Highlights that laser treatment parameters should be adjusted for different skin groups, but without explicit melanin or pigmentation quantification, it may overlook intra-group variability and other factors affecting phototype assignment, potentially limiting precision in dosing.
Denstedt et al. (2014) [44]	To extract spectral features from hyperspectral imaging of tissue and correlate them with physical parameters such as melanin concentration, oxygenation, and blood volume.	0.5% and 6% melanin concentrations.	Limited skin type range may overlook effects of non-homogeneous or higher melanin distributions on the hyperspectral signals.
Huang et al. (2015) [60]	Reduce measurement artefacts caused by melanin concentration, scattering, and blood absorption using a multispectral imaging algorithm for noncontact and quantitative assessment of cutaneous tissue oxygen saturation.	Melanin concentrations: 2% (Caucasians), 13% (Asians), 30% (Africans).	Assigning fixed concentrations to broad ethnic categories assumes uniform pigmentation within each group, neglecting intra-population variability
Zhao et al. (2016) [29]	Model in vivo Raman spectroscopy of skin and analyse how light propagates and is absorbed in different layers of skin, particularly in the epidermis and dermis. Relevant to skin cancer diagnosis.	Light skin.	Could provide a baseline spectrum and reduce interference from low melanin levels to improve signal-to-noise and layer-specific analysis in the epidermis and dermis. Likely to bias the understanding of photon penetration, scattering, and Raman band intensities in highly pigmented skin, potentially affecting calibration of diagnostic thresholds for skin cancer in darker individuals.
Ghassemi et al. (2016) [37]	Investigate the effect of melanin content on laser-induced heating in breast cancer imaging.	Melanin concentrations: 1% (Type II—Light skin), 13.5% (Type IV—moderate skin), 30.5% (Type VI—dark skin).	Discrete melanin levels enables controlled comparison of laser-induced heating but introduces stepwise rather than continuous changes in optical absorption, which can mask nonlinear relationships between melanin and thermal response. Melanin % intervals should be smaller for such applications.
Akaho et al. (2017) [34]	Analyse the relationship between dark circles and chromophore concentrations (e.g., melanin, haemoglobin) for cosmetic applications.	1–10% melanin concentration.	Confined range potentially biases cosmetic assessment and product design to light and fairly moderate individuals. Such applications require more detail when classifying pigmentation levels including hues, undertones, etc.
Mustafa et al. (2017) [58]	Analyse how melanin and hydration affect near-infrared (NIR) reflectance for body fat measurement in neonates.	Melanin concentrations: 1% and 30% = Caucasian and African skin, respectively.	With melanin effects being relatively weak in comparison to water attenuation in the NIR, the influence of pigmentation on signal depth and reflectance may be masked in retrieved body fat measurements when using two single extreme discrete values. Continuous melanin variation can be modelled to capture subtle effects of photon behaviour.
Burns et al. (2018) [53]	Evaluate light absorption at different melanin levels and explore ways to improve laser therapy outcomes for darker skin tones.	Melanin concentrations: 4%, 15%, and 50% = Lightly, moderately, and heavily pigmented skin.	Fixed concentrations assume uniform epidermal melanin and ignore non-homogeneous distributions, melanosome size effects, and individual variability in skin thickness and vascularisation, especially in heavily pigmented skin.
Li et al. (2018) [57]	Develop an optimised version of the Kubelka–Munk Genetic Algorithm (KMGA) for fast multispectral skin lesion assessment.	1–43% melanin concentrations.	Intra-lesion heterogeneity or variations in lesion vs. surrounding skin may not be captured, potentially limiting accuracy in lesion classification and spectral feature extraction.
Hung et al. (2019) [28]	Track how UV light interacts with skin layers and calculates the absorbed UV power at different hair follicle depths.	Caucasian skin types I–II.	Only tracks UV penetration and absorption in low-melanin skin, which is not indicative of follicular UV exposure and photodamage risk in other ethnic groups due to differences in hair follicle density, diameter, and pigmentation.
Chatterjee & Kyriacou (2019) [6]	Analyse light–tissue interactions in reflectance and transmittance PPG.	10% melanin concentration.	Accounts for melanin effects on the PPG signal in both modalities, but assumes homogeneous epidermal layers which does not represent real skin physiology.
Verdel et al. (2019) [45]	Combining Pulsed Photothermal Radiometry (PPTR) and Diffuse Reflectance Spectroscopy (DRS) with Monte Carlo modelling to assess optical and physiological properties of skin such as melanin concentration, blood content, etc.	1.3–1.9% melanin concentrations (Caucasian skin).	Narrow melanin concentration range enables PPTR and DRS signals to be attributed primarily to epidermal melanin and blood content with minimal absorption confounding. Limits understanding of how higher melanin levels alter photon transport, heat deposition, and signal amplitude, potentially reducing the accuracy and applicability of Monte Carlo-based parameter extraction for more pigmented populations.
Naglič et al. (2019) [63]	Monitoring physiological changes in skin reflectance from melanin variations due to sun exposure.	No exact range specified. Example values from figures suggest melanin concentrations of around 0.5% and 1.5%.	Focusing on very light skin implies that the reflectance measurements are more sensitive to burning-related changes, making the study more suited to erythema and acute sun response, and less to long-term pigmentation changes in moderate-to-dark skin.
Zhang et al. (2019) [64]	A correction model to account for melanin’s inhomogeneous distribution for reflectance spectroscopy in heavily pigmented skin.	No exact range specified but simulates dark skin types IV–VI.	Layer model for non-homogeneous melanin distribution improves extraction of reliable tissue optical properties in darkly pigmented skin.
Chatterjee et al. (2020) [30]	Investigate the origins of the PPG signal by focusing on the impact of absorbance, reflectance, and penetration depth at multiple wavelengths and different skin layers.	10% melanin concentration.	Establishes reference PPG signals for a given skin type, which can later be compared against other pigmented group models (also modelled homogeneously) to quantify melanin effects systematically, i.e., by using correction factors.
Ying et al. (2020) [74]	Investigate the effect of melanin distribution on laser therapy for port wine stain treatment and improve parameter selection for laser thermotherapy.	10 distinct melanin distribution profiles based on melanin formation and migration.	Non-uniform distribution of melanin models local hotspots of absorption when exposed to laser light, even if average melanin content is the same. Must be accounted for in laser parameter selection or could lead to poor clinical outcomes.
Hernández-Quintanar et al. (2020) [61]	Improve accuracy of pulse oximetry measurements by accounting for skin thickness and melanin content.	Melanin concentrations: 3.5% (lightly pigmented skin), 13.5% (moderately pigmented skin), 30.5% (strongly pigmented skin).	Captures structural differences between skin groups, reflecting how both epidermal and dermal layer depth influence photon pathlength, absorption, and scattering.
Robbins et al. (2021) [41]	Enabling non-invasive monitoring of tumour response to chemotherapy in patients with different skin tones.	Absorption coefficients of 0.01–2 ${mm}^{-1}$ are simulated to cover light to dark skin types. Instead of defining melanin as a fixed percentage, the study estimates melanin content dynamically using a melanin index (MI).	MI accounts for inter-individual variability and temporal changes in skin pigmentation overtime. Enhances robustness of optical tumour monitoring across diverse ethnic groups.
Boonya-Ananta et al. (2021) [43]	Evaluates how skin tone and obesity affect the accuracy of PPG-based heart rate sensors in Apple Watch S5, Fitbit Versa 2, and Polar M600.	Melanin concentrations: 3% (Type I), 10% (Type II), 16% (Type III), 23% (type IV), 32% (Type V), and 42% (Type VI).	Melanin groupings enable direct comparison of sensor performance across individual phototypes. However, population intra-type variability limits generalisability across diverse population consumers.
Dremin et al. (2021) [52]	Assess how melanin concentration affects LDF and tissue reflectance oximetry measurements.	1–45% melanin concentrations, from very light to very dark skin.	Uniform distribution of melanin may oversimplify photon scattering and absorption relevant to LDF signal formation.
Colas et al. (2021) [59]	Quantify skin layer contributions in light transport simulations to improve non-invasive skin cancer diagnostics and estimate optical properties of different skin layers.	Melanin concentrations: 1% (Phototype I, very fair), 4% (Phototype II, fair), 8% (Phototype III, moderately fair), and 11% (Phototype IV, dark).	The low value of 11% for Phototype IV (compared to similar studies) likely underestimates epidermal absorption and scattering, potentially biasing simulated photon propagation and the inferred optical properties of darker skin layers.
Fine et al. (2022) [40]	Investigate the effect of age, skin tone, and device wavelength on PPG signal strength and feature extraction for blood pressure estimation.	3–30% melanin concentrations.	Uniform melanin assumptions and age-dependent epidermal thickness are likely to misrepresent local variations in absorption, affecting derivative features like AC/DC ratios or pulse waveform characteristics used for blood pressure estimation.
Althobaiti (2022) [46]	Optimise a dual-channel near-infrared (NIR) glucose sensor for different skin colours for signal-to-noise ratio (SNR) and dermis sensitivity.	Melanin concentrations: 2%, 10%, 20%, and 30%, representing various skin tones from light to dark.	Regional pigmentation differences may still introduce deviations from SNR model predictions.
Nishidate (2022) [49]	Estimation of blood oxygen saturation and haemoglobin concentration in skin using RGB measurements.	1% to 10% melanin concentrations.	Shorter wavelengths are disproportionately attenuated by higher melanin levels, skewing the ratios and introducing systematic bias. For skin > 10% melanin, RGB signals would be increasingly dominated by epidermal absorption in comparison to blood absorption, which compromises the accuracy of SpO_2_ and haemoglobin estimates.
Harrison-Smith et al. (2022) [56]	Investigate and reduce racial bias in transcutaneous bilirubin and pulse oximetry measurements.	Light, mixed, and dark skin. No exact values specified.	Using broad skin categories without specifying pigmentation metrics highlights general trends in racial bias but limits quantitative interpretation, especially with confounding bilirubin levels on SpO_2_ output.
Hou et al. (2022) [65]	Investigates how optical property changes (due to ischemia and hyperemia) affect NADH fluorescence measurements.	Melanin concentrations: 2% (Caucasian), 13% (Asian), 30% (African).	As with the Fitzpatrick scale, assigning ethnicities to melanin levels can be problematic as these categories are social constructs, not previse biological measurement. Using them as proxies for melanin content can misrepresent actual skin optical properties in single and multi-ethnic populations.
Arefin et al. (2022) [67]	Investigate racial bias in pulse oximeter calibration by assessing how different racial enrolment distributions impact SpO_2_ calibration accuracy.	Melanin concentration is stochastically sampled along with other chromophores (blood, bilirubin) to create 1200+ skin pigmentation models.	Modelling inter-individual differences in pigmentation is likely to increase sensitivity of combined chromophore absorption by pulse oximeters, potentially improving device calibration across diverse populations.
Al-Halawani et al. (2022) [70]	Explores the effect of melanin concentration in the epidermis at different wavelengths in the visible range in reflective geometry.	Melanin concentrations: 5%, 10%, 15%, 20%.	Provides preliminary insights into reflectance sensitivity of superficial skin layers in isolation to other chromophores. Should capture local heterogeneity for more in-depth understanding of melanin effects.
Chen et al. (2023) [39]	To correct melanin and haemoglobin absorption in a non-invasive method to measure bilirubin concentration in adults for liver monitoring.	$\mathrm{Absorption}\;\mathrm{coefficients}\;\mathrm{of}\;0.01\;\mathrm{to}\;2\;{mm}^{-1}$ are simulated to cover light to dark skin types.	Broad coefficient range improves applicability across diverse skin tones, especially for the integration of personalised calibration or adaptive modelling in non-invasive optical diagnostics. Could model melanin non-homogeneously by assigning optical properties to individual voxels.
Bolic (2023) [62]	Analyse the effects of melanin concentration and air gap depth on the accuracy of reflectance-mode pulse oximeters.	Melanin concentrations: 0.3%, 3%, 8%, 12%, and 16%.	Deviations in the simulated calibration curve for 8% melanin concentration may not reflect true tissue behaviour but rather limitations in model sampling or parameterisation, emphasising the importance of validating models, particularly across a higher melanin range.
Else et al. (2024) [48]	Investigate how skin melanin concentration affects photoacoustic imaging (PAI) and blood oxygenation measurements.	2% to 40% melanin concentrations (Skin Types I–VI).	Could incorporate spectrally resolved absorption and scattering coefficients that vary with both wavelength and concentration to retrieve more accurate depth and wavelength-dependent signal changes.
Larsson et al. (2024) [55]	Estimate blood oxygen saturation in real time from multispectral imaging for clinical applications such as ischemia detection, microcirculatory studies, and wound healing assessment.	Melanin molarity (μM) is randomised. No exact values specified.	Increases sensitivity of SpO_2_ estimates to skin heterogeneity, enhancing detection of subtle physiological changes. May require more advanced signal processing to mitigate stochastic noise from random sampling and ensure clinical accuracy.
Narayana Swamy et al. (2024) [68]	Provide solutions for overestimated oxygen saturation in heavily pigmented skin types via pulse oximeter design.	5% and 25% melanin concentrations (Light (Type II) and dark (Type V), respectively).	The potential nonlinear behaviour of moderate and darker skin types with SpO_2_ is particularly important for device modifications since they often co-vary with epidermal thickness, scattering properties, and melanosome size.
Al-Halawani et al. (2024) [69]	Simulates the effect of light, moderate, and dark skin on key optical parameters in transmittance and reflectance finger photoplethysmography.	Melanin concentrations: 2.55%, 15.55, and 30.5%.	Accounts for light, moderate, and dark skin types. However, uniform melanin assumptions do not capture spatial heterogeneity, which may differentially affect transmittance vs. reflectance measurements.
Al-Halawani et al. (2024) [71]	Simulates the effect of light, moderate, and dark skin on pulse oximeter calibration curves in transmittance mode.	Melanin concentrations: 2.55%, 15.55, and 30.5%.	Establishes trends in calibration algorithms for discrete skin groups, but requires continuous, stochastic, or layered pigmentation modelling for more inclusive calibration across diverse populations.
Reiser et al. (2025) [47]	Assess signal loss due to melanin absorption, compare different calibration models, and suggest improvements for skin colour-adapted pulse oximeter calibration	Melanin concentrations: 2.55%, 5.5%, 10.5%, 15.5%, 20.5%, 25.5%, and 30.5%, representing various skin tones from light to dark.	Smaller incremental changes in melanin concentration captures more subtle changes in oximeter output, potentially easing data interpretation. Static melanin assumption continuous to oversimplify the extent of adaptive calibration measures required for inclusive monitoring of SpO_2_.
Al-Halawani et al. (2025) [73]	Analyses intensity changes from Monte Carlo-simulated reflectance PPG signals across light, moderate, and dark skin types at oxygen saturations of 70% and 100%.	Optical properties of light, moderate, and dark epidermis are obtained spectroscopically.	Approach for pigmentation modelling shifts from arbitrary melanin % to a more physical optical representation of absorption and scattering, which better captures how epidermal pigmentation influences photon transport.
Al-Halawani et al. (2025) [72]	Simulates the effect of light, moderate, and dark skin on pulse oximeter calibration curves in reflectance mode.	Melanin concentrations: 2.55%, 15.55, and 30.5%.	Enables direct comparison with previous study [72]; however, encounters the same drawbacks of the pigmentation modelling approach.

**Table 3:** Comparison between calculated and spectroscopy-based melanin absorption coefficients at different wavelengths.

Wavelength (nm)	532	755	1064
$\mu_{a}melanin$ from the literature [77] [${mm}^{-1}$]	55.5	16.3	5.0
$\mu_{a}melanin$ from calculations (Equation (13)) [${mm}^{-1}$]	55.2	17.2	5.5

**Table 4:** Optical properties of the epidermis extracted from [40].

		515 nm	660 nm	880 nm
$\mu_{a}$[${mm}^{-1}$]	$v_{mel}$ = 3%	0.196	0.086	0.033
	$v_{mel}$ = 10%	0.628	0.275	0.106
	$v_{mel}$ = 20%	1.243	0.544	0.209
	$v_{mel}$ = 30%	1.858	0.813	0.313
$\mu_{s}$[${mm}^{-1}$]		38.84	30.30	22.73

**Table 5:** Optical properties of the epidermis extracted from [77].

		585 nm	595 nm	810 nm	940 nm	1064 nm
$\mu_{a}$[${mm}^{-1}$]	Light	6.071	5.738	2.065	1.266	0.845
	Moderate	20.15	19.045	6.824	4.162	2.759
	Heavy	38.252	36.153	12.944	7.885	5.220
$\mu_{s}$[${mm}^{-1}$]		15.606	14.869	14.802	10.527	8.137
g		0.8	0.8	0.91	0.91	0.91

## Data Availability

No new data was created or analysed in this review.
